# Barley β-glucan improves metabolic condition via short-chain fatty acids produced by gut microbial fermentation in high fat diet fed mice

**DOI:** 10.1371/journal.pone.0196579

**Published:** 2018-04-26

**Authors:** Junki Miyamoto, Keita Watanabe, Satsuki Taira, Mayu Kasubuchi, Xuan Li, Junichiro Irie, Hiroshi Itoh, Ikuo Kimura

**Affiliations:** 1 Department of Applied Biological Science, Graduate School of Agriculture, Tokyo University of Agriculture and Technology, Fuchu-shi, Tokyo, Japan; 2 AMED-CREST, Japan Agency for Medical Research and Development, Chiyoda-ku, Tokyo, Japan; 3 Department of Internal Medicine, School of Medicine, Keio University, Shinjuku-ku, Tokyo, Japan; University of Hawai'i at Manoa College of Tropical Agriculture and Human Resources, UNITED STATES

## Abstract

Dietary intake of barley β-glucan (BG) is known to affect energy metabolism. However, its underlying mechanism remains poorly understood because studies have presented inconsistent results, with both positive and negative effects reported in terms of satiety, energy intake, weight loss, and glycemic control. The objective of this study was to clarify the physiological role underlying the metabolic benefits of barley BG using a mouse model of high fat diet (HFD)-induced obesity. Male 4-wk-old C57BL/6J mice were fed an HFD with 20% barley flour containing either high BG (HBG; 2% BG) or low BG (LBG; 0.6% BG) levels under conventional and germ-free (GF) conditions for 12 wks. In addition, mice were fed either an HFD with 5% cellulose (HFC; high fiber cellulose) or 5% barley BG (HFB; high fiber β-glucan) for 12 wks. Then, metabolic parameters, gut microbial compositions, and the production of fecal short-chain fatty acids (SCFAs) were analyzed. The weight gain and fat mass of HBG-fed mice were lower than those of control mice at 16-wk-old. Moreover, the secretion of the gut hormones PYY and GLP-1 increased in HBG-fed mice, thereby reducing food intake and improving insulin sensitivity by changing the gut microbiota and increasing SCFAs (especially, butyrate) under conventional condition. These effects in HBG-fed mice were abolished under GF conditions. Moreover, the HFB diets also increased PYY and GLP-1 secretion, and decreased food intake compared with that in HFC-fed mice. These results suggest that the beneficial metabolic effects of barley BG are primary due to the suppression of appetite and improvement of insulin sensitivity, which are induced by gut hormone secretion promoted via gut microbiota-produced SCFAs.

## Introduction

Diet is the most important energy resource for daily activities. However, the dysregulation of energy homeostasis caused by excess and unbalanced diets leads to metabolic disorders, such as obesity and type 2 diabetes [1]. Obesity has recently become a serious public health problem worldwide owing to its increasing prevalence and contribution to various disease complications such as metabolic syndrome [1, 2]. Obesity progression occurs due to a long-term imbalance between energy intake and utilization, which then affect multiple signaling pathways via metabolites and hormones [3]. Excess food energy intake, especially through high levels of fat and carbohydrates, insufficient exercise, and genetic factors are considered risk factors for the development of obesity [4].

Whole-grain foods, which contain the endosperm, germ, and bran of seeds, have been reported to improve insulin sensitivity and reduce obesity, while they have also been found to be associated with metabolic syndrome in adults [5]. In epidemiological studies concerning the metabolic benefits of whole-grain foods, dietary fiber is considered to be the most important factor in explaining the health effects of the diet [5]. Based on its water solubility, dietary fiber is roughly divided into soluble and insoluble fiber. Fiber is known to have stronger physiological effects on the improvement of bowel disorders such as constipation [6, 7]. The laxative effect of dietary fiber arises from the mechanical irritation of the gut mucosa by some insoluble fiber and the consequent secretion of water and mucous. This mechanism requires that the fiber resists fermentation and remain relatively intact throughout the large bowel so as to increase stool water content and form bulky soft easy-to-pass stools. Due to its viscosity, fiber also exhibits reduced postprandial plasma glucose and improved insulin sensitivity [8]. This is presumed to be due to an increase in the viscosity of the gastro-intestinal contents, which reduces the gastric emptying rate and the absorption and transport of digested nutrients [9, 10]. In addition, since the soluble fiber escapes host digestion and reaches to colon, they also have properties producing short-chain fatty acids (SCFAs), such as acetate, propionate, and butyrate, by stimulating gut microbial fermentation [11]. SCFAs, in addition to being involved in de novo synthesis of lipids and serving as energy sources for the host [12], play an essential role as signaling molecules in host energy homeostasis-related physiological functions, such as gut hormone secretion via the G protein-coupled receptors GPR41 or GPR43 [13–18].

β-glucan (BG) is found in cereal grains and contained in the cell walls of oat and barley endosperms [19, 20]. Barley and oat BGs are a soluble fiber that is highly viscous in solution [19, 20]. Barley or oat BG consumption by human participants has produced inconsistent results in term of its metabolic benefits on satiety, energy intake, weight loss, and insulin sensitivity [21–27]. This is likely because the effects of cereal grains differ based on the supplemented amounts and sources of BG. A diet containing high levels of barley BG has been shown to produce metabolic benefits in terms of postprandial insulin and glucose responses [21–23]. However, the detailed physiological role underlying the metabolic benefits of barley BG remain unclear.

In this study, we investigated the beneficial metabolic effects of barley flour containing varying amounts of BG in a mouse model of high-fat diet (HFD)-induced obesity. In particular, we studied the relationship between the gut microbiota and SCFA metabolites by assessing their effects in barley flour-fed germ-free (GF) mice. We confirmed that barley BG at least causes appetite suppression and improved insulin sensitivity via SCFA-induced production of gut hormones.

## Results

### Barley flour containing high levels of BG suppresses HFD-induced obesity

**Fig 1:**
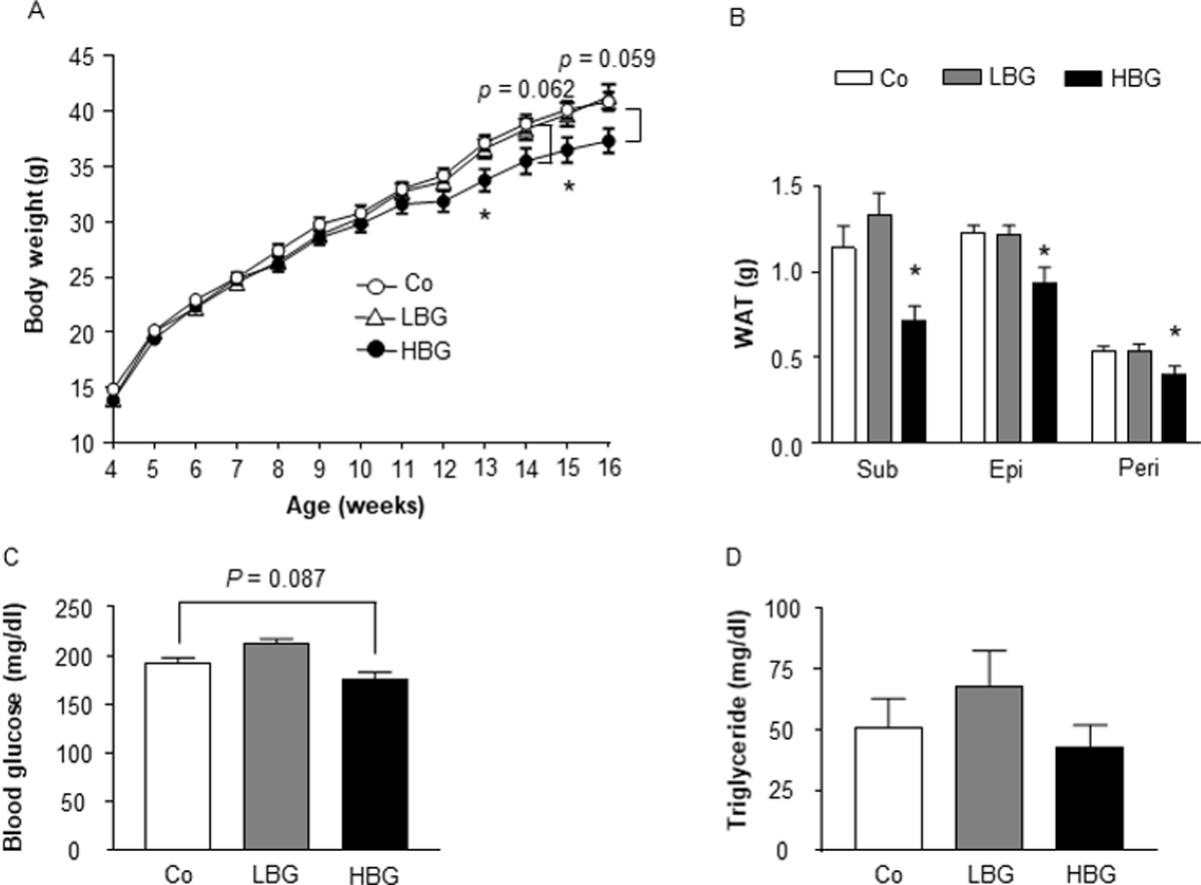
HBG barley flour suppresses HFD-induced obesity. Body weight changes (A), fat mass (B), blood glucose (C), and plasma triglyceride (D) were measured in male mice fed Co, HBG, or LBG diets for 12 weeks. Values are means ± S.E.M. n = 8–13. *, *P* < 0.05, compared with Co (Tukey-Kramer test). Co, control; Epi, epididymal; HBG, β-glucan rich barley; LBG, general barley; Peri, perirenal; Sub, subcutaneous; WAT, white adipose tissue.

We first investigated changes in metabolic parameters following barley flour feeding in a mouse model of HFD-induced obesity. In this experiment, 4-wk old mice were fed HFDs containing 20% barley flour with high levels of BG (HBG; 2% BG) or low levels of BG (LBG; 0.6% BG) for 12 weeks (S1 Table). Body weights were comparable between control and LBG-fed mice but were significantly lower in HBG-fed mice during growth (Fig 1A). The fat mass of white adipose tissue (WAT) was also comparable between control and LBG-fed mice but was significantly lower in HBG-fed mice at 16 weeks of age (Fig 1B). Moreover, the plasma glucose levels of HBG-fed mice tended to be lower than those of control mice (Fig 1C; *P* = 0.087). However, triglyceride levels were similar between control and HBG-fed mice and were slightly higher in LBG-fed mice (Fig 1D). Therefore, the HBG diet suppressed the elevation of plasma glucose level and the increase in body fat mass, preventing the HFD-fed mice from becoming obese.

### HBG barley flour suppresses appetite and improves insulin sensitivity

**Fig 2:**
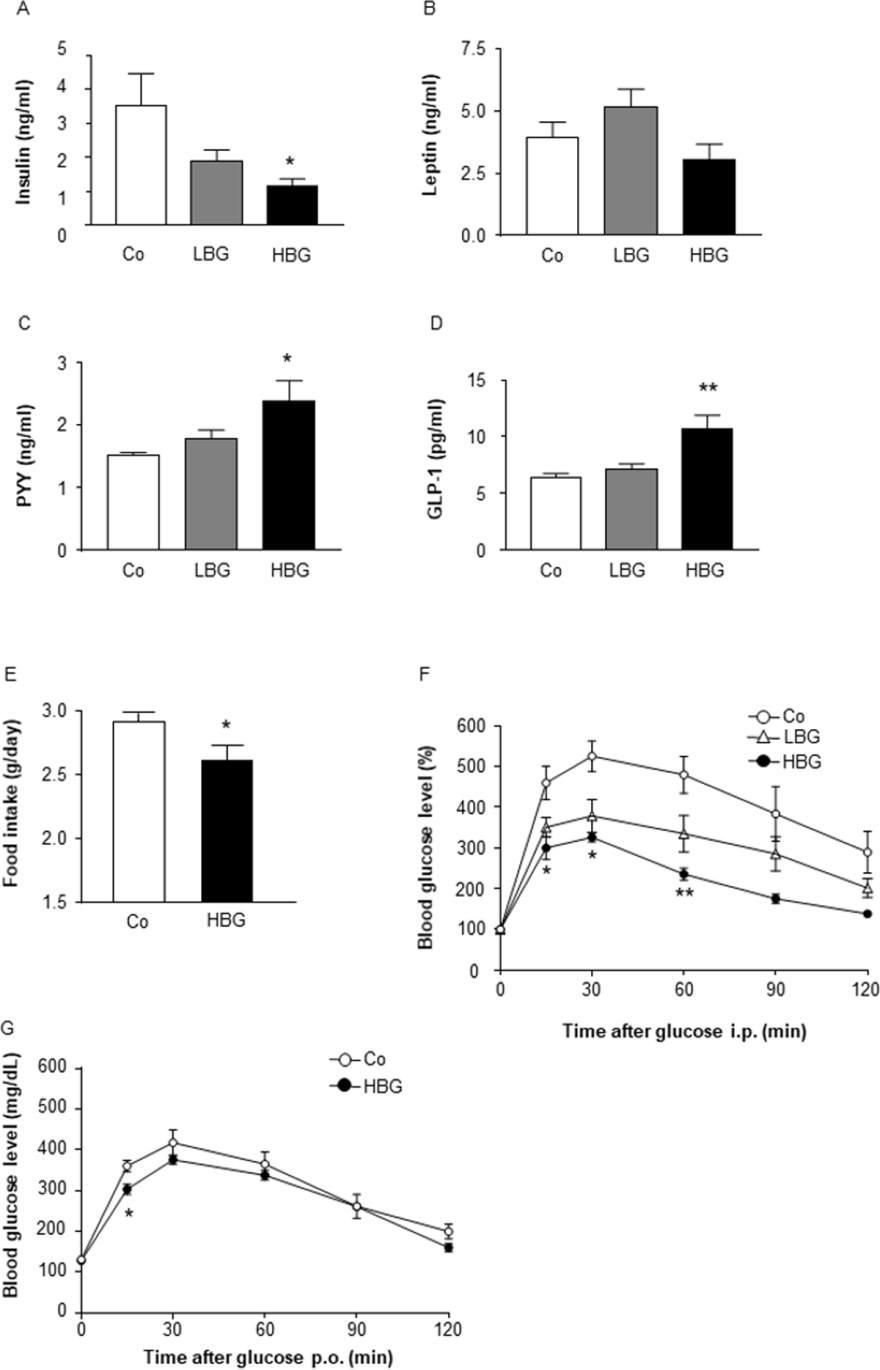
HBG barley flour suppresses appetite and improves insulin sensitivity. Plasma insulin (A), leptin (B), PYY (C), and GLP-1 levels (D) were measured in male mice fed Co, HBG, or LBG diets for 12 weeks (n = 8–10). Daily food intakes were measured at 10 weeks of age (E), and glucose tolerance test was investigated at 14 (ipGTT; F) and 15 (OGTT; G) weeks of age in male mice fed Co, HBG or LBG diet. Values are means ± S.E.M. n = 4–6. *, *P* < 0.05, and ** *P* <0.01, compared with Co (Tukey-Kramer test for A-D, and F) (student’s t-test for E and G). Co, control; HBG, β-glucan rich barley; LBG, general barley; GLP-1, glucagon-like peptide 1; PYY, peptide YY.

We next examined changes in circulating plasma hormones related to energy metabolism following HBG feeding. Plasma insulin levels of HBG -fed mice were significantly lower than those of control mice (Fig 2A), whereas plasma leptin levels were comparable among the three groups (Fig 2B). Moreover, levels of the plasma peptide YY (PYY) were significantly higher in HBG-fed mice than in control mice (Fig 2C). Similarly, plasma glucagon-like peptide-1 (GLP-1) levels were significantly higher in HBG-fed mice than in control mice (Fig 2D). Thus, HBG-fed mice exhibited lower levels of plasma insulin and higher levels of plasma gut hormones. The gut hormone PYY is an anorexigenic hormone that directly suppresses appetite in the brain, inhibits gut motility, increases intestinal transit rate, and reduces the harvest of energy from the diet [14]. The gut hormone GLP-1 acts systemically to increase pancreatic b-cell growth, glucose-dependent insulin secretion, and satiety via the hypothalamus, as well as decreasing glucagon secretion [15]. Therefore, to clarify the influence of the HBG diet on physiological functions, we further examined food intake and postprandial glycemic changes. As expected, the food intake in HBG-fed mice was significantly lower than that in control mice (Fig 2E). Moreover, HFD-induced impaired glucose tolerance, was significantly attenuated in HBG-fed mice compared with that in control mice (Fig 2F and 2G). Thus, HBG feeding suppressed the appetite and improved insulin sensitivity by increasing plasma levels of PYY and GLP-1, thereby inducing resistance to obesity.

### Intake of barley flour modifies the gut microbial composition and enhances the production of SCFAs

**Fig 3:**
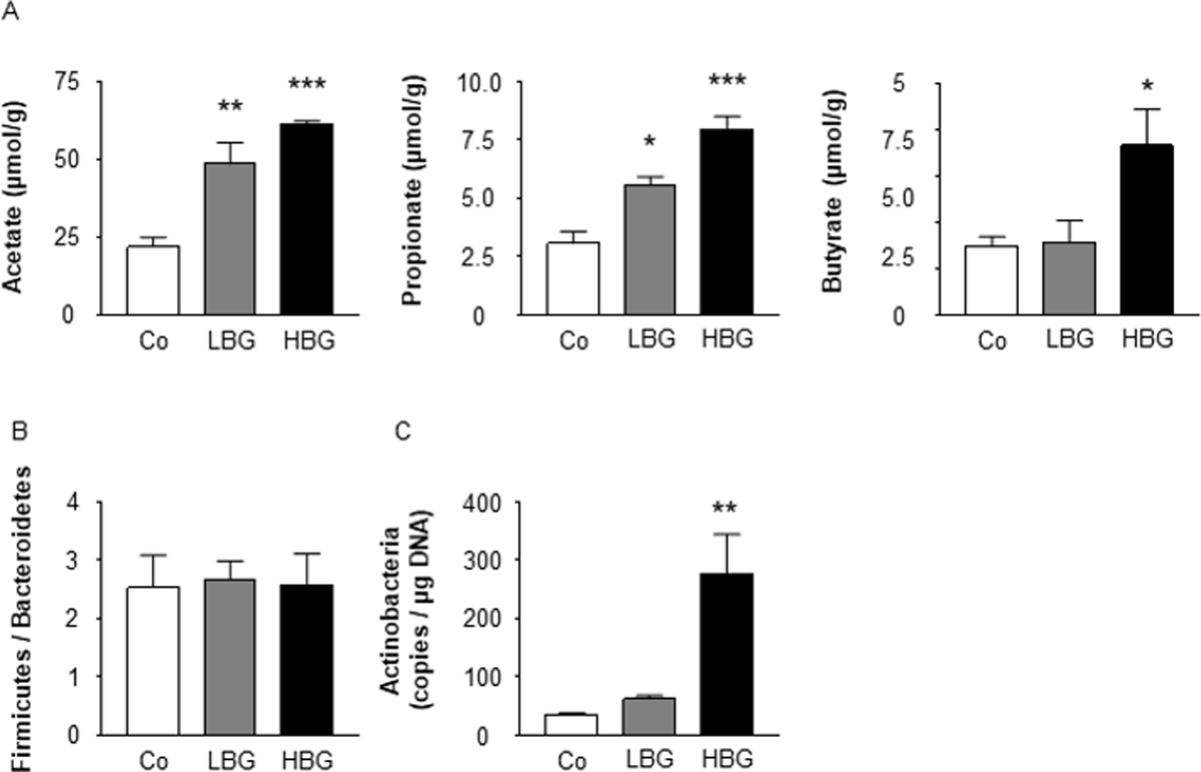
HBG barley flour produces SCFAs and changes the gut microbial composition. Fecal short chain fatty acids were measured in male mice fed Co, HBG, or LBG diets for 12 weeks (A). *Firmicutes* / *Bacteroidetes* ratio (B), and *Actinobacteria* (C) in feces were measured by quantitative real-time PCR for Co, HBG, or LBG diets for 2 weeks. Values are means ± S.E.M. n = 4–8. *, *P* < 0.05, **, *P* < 0.01, and ***, *P* < 0.001, compared with Co. Co (Tukey-Kramer test), control; HBG, β-glucan rich barley; LBG, general barley.

The gut microbiota affects host homeostasis through the digestion and fermentation of indigestible polysaccharides such as dietary fiber. SCFAs are produced via the gut microbial fermentation of dietary fiber, including BG [13]. These SCFAs promote the secretion of gut hormones such as GLP-1 and PYY from colonic enteroendocrine cells. Therefore, we next examined the effects of a HBG diet on the gut microbiota of HFD-fed mice. Levels of fecal acetate, propionate, and butyrate, the main SCFAs (especially, butyrate) produced by the gut microbiota, markedly increased in HBG- and LBG-fed mice (Fig 3A). We also examined the gut microbial compositions of the three groups. Firmicutes and Bacteroidetes are the two dominant phyla in the gut, and many studies have found a relationship between a higher Firmicutes/Bacteroidetes ratio and an increased amount of SCFAs in the feces [28, 29]. Although the Firmicutes/Bacteroidetes ratio was similar between HBG-/LBG-fed and control mice (Fig 3B), the abundance of Actinobacteria that encompasses the genus *Bifidobacterium*, which is a major acetate producer, was significantly higher in both HBG- and LBG-fed mice than in the control (Fig 3C). Thus, barley flour induced the production of SCFAs by increasing the abundance of Actinobacteria.

### Metabolic benefits of barley flour are abolished under GF conditions

**Fig 4:**
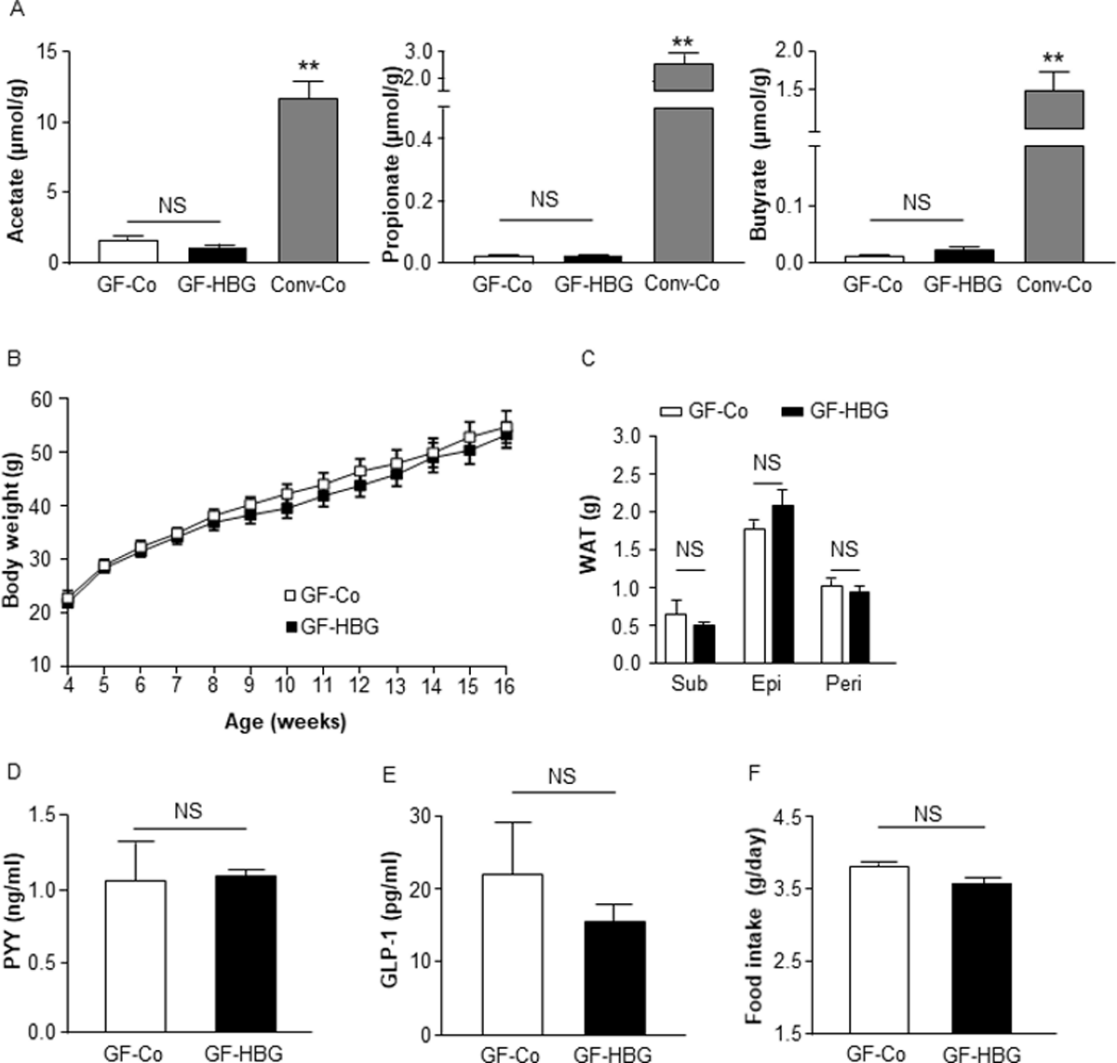
Metabolic benefits of HBG barley flour are abolished under GF conditions. Fecal short chain fatty acids in germ-free and conventional mice were measured by GC/MS (A). Body weight changes (B), fat mass (C), plasma PYY (D), and GLP-1 levels (E) were measured in male germ-free mice fed a Co or HBG diets for 12 weeks. Daily food intakes were measured at 8 to 10 weeks of age (F). Values are means ± S.E.M. n = 5. **, *P* < 0.01, compared with Co (Tukey-Kramer test for A) (student’s t-test for B-F). Co, Control; Conv-Co, Conventional mice fed a Co diet; Epi, epididymal; GF-Co, Germ-Free mice fed a Co; GF-HBG, Germ-Free mice fed a HBG; GLP-1, glucagon-like peptide 1; HBG, β-glucan rich barley; Peri, perirenal; PYY, peptide YY; Sub, subcutaneous; WAT, white adipose tissue.

To determine whether the metabolic benefits of an HBG diet depends on the gut microbiota, we further assessed the effects of the HBG diet on mice under GF conditions. Under GF conditions, levels of fecal SCFAs were hardly detectable in both control and HBG-fed mice (Fig 4A). Body weight and fat mass were also comparable between control and HBG-fed GF mice (Fig 4B and 4C). In addition, levels of the plasma gut hormones PYY and GLP-1 were similar between control and HBG-fed GF mice (Fig 4D and 4E), as was food intake (Fig 4F). Thus, the metabolic benefits of barley flour depend upon the gut microbial fermentation of BG.

### Barley flour with 5% BG results in metabolic effects similar to those of the HBG diet

**Fig 5:**
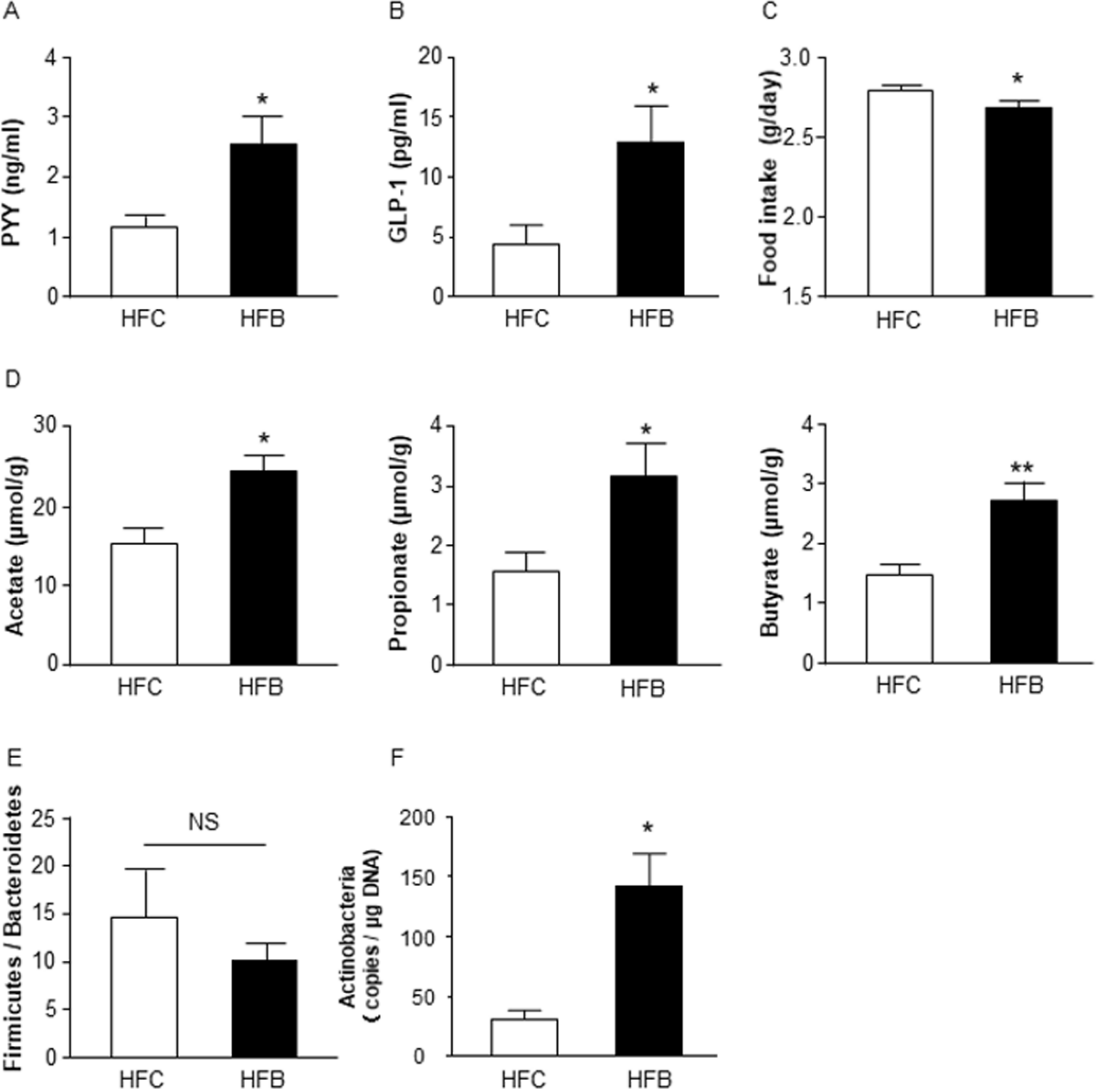
HFB suppresses HFD-induced obesity. Plasma PYY (A) and GLP-1 levels (B) were measured in male mice fed HFC or HFB for 12 weeks. Daily food intakes were measured at 9 to11 weeks of age (C). Fecal short chain fatty acids were measured by GC/MS (D). *Firmicutes* / *Bacteroidetes* ratio (E) and *Actinobacteria* (F) in feces were measured by quantitative real-time PCR. Values are means ± S.E.M. n = 7–9. *, *P* < 0.05, and **, *P* < 0.01, compared with HFC (student’s t-test). Epi, epididymal; GLP-1, glucagon-like peptide 1; HFB, high fat diet + 5% barley β-glucan; HFC, high fat diet + 5% cellulose; Peri, perirenal; PYY, peptide YY; Sub, subcutaneous; WAT, white adipose tissue.

Finally, to examine whether the beneficial metabolic effects of barley flour are attributed to BG, we compared the effects of an HFD containing 5% BG (HFB) to those of an HFD containing 5% cellulose (HFC) as control (S2 Table) for 12 weeks. Similar to HBG-fed mice, the plasma PYY levels of HFB-fed mice were significantly higher than those of HFC-fed mice at 16 weeks (Fig 5A). Plasma GLP-1 levels were also significantly higher than in control mice (Fig 5B). Moreover, the food intake in HFB-fed mice was significantly lower than that in HFC-fed mice (Fig 5C). Fecal levels of acetate, propionate, and butyrate were also higher in HFB-fed mice (Fig 5D). The gut microbial compositions (ratio of Firmicutes to Bacteroidetes) were similar between HFB- and HFC-fed mice (Fig 5E), whereas as in HBG-fed mice, the abundance of Actinobacteria was significantly elevated in HFB-fed mice compared with that in HFC-fed mice (Fig 5F). Thus, it is clear that BG is primarily responsible for the metabolic benefits of barley flour.

## Discussion

Barley BG has been reported to improve insulin resistance and obesity. However, the molecular mechanisms underlying this effect remain unclear. In this study, we found that barley flour containing high levels of BG was efficacious against weight gain and fat increases by reducing food intake and improving insulin sensitivity. These metabolic benefits of barley BG were associated with the SCFAs produced by the gut microbiota and their promoting effects on gut hormone secretion.

Our work using an HFD containing high levels of BG (HBG: 2–4% BG) confirmed the earlier results that support the metabolic benefits of barley flour [30, 31], though 3% BG was previously reported to confer no improvement in glucose tolerance [32]. These discordant results may reflect differences in experimental conditions (e.g. the period of treatment and the source of BG). Specifically, in line with our study, positive effects of BG on metabolism have been reported in long-term treatment in mice [30, 31], whereas short-term treatment (2 weeks) with BG contributed to negative effects on metabolism in rats [32]. Although the LBG diet also increased levels of fecal SCFAs and gut hormones, it did not exert anti-obesity effects. Rather surprisingly, the LBG diet tended to increase body weight and plasma glucose and triglyceride levels, indicating that other components in barley flour might influence the regulation of energy harvesting, as the gut hormone production induced by the LBG diet was minimal. Elucidation of physiological functions of barley flour via other components beside b-glucan are needed to furthermore examine.

We found that barley BG led to increased levels of plasma PYY and GLP-1. PYY and GLP-1 suppresses the appetite, and GLP-1 promotes glucose-dependent insulin secretion [33, 34]. Therefore, we speculate that the suppression of food intake and improvement in insulin sensitivity induced by the barley flour diets were due to the promotion of gut hormone secretion from enteroendocrine cells by SCFAs. The mechanism of SCFA-stimulated gut hormone secretion likely involves the SCFA receptors GPR41 and GPR43 [14, 15]. In the future, studies of mice deficient in these SCFA receptors will provide insight into the role of barley BG in gut hormone-related metabolic signaling and physiological functions. Moreover, SCFA-stimulated gut hormone secretion from enteroendocrine cells has been demonstrated not only in mice studies but also in human studies [33]. Hence, the results obtained in mice may be applicable to human studies and the metabolic benefit conferred by barley b-glucan as observed in mice may be reproducible in humans.

Both of HFDs containing 20% barley flour and 5% barley BG changed the gut microbial composition in mice. In particular, the abundance of the phylum Actinobacteria increased dramatically in HBG- and HFB-fed mice compared with that in control mice. It is known that in addition to Phylum Bacteroidetes and Firmicutes, Phylum Actinobacteria also produces SCFAs by fermenting indigestible polysaccharides [35]. Therefore, these gut microbes may have an important role in SCFA production via the fermentation of barley BG. Additionally, it has been reported that the presence of *Prevotella copri* in barley kernel-based bread affects host glucose metabolism and suppresses an increase in postprandial glucose [36]. However, in our study, the Firmicutes to Bacteroidetes ratio did not change as a result of either the HBG or HFB diets. This may be due to the fact that our studies assessed the difference of the effects between the long-term BG administration in mice and the short-term BG administration in humans.

In this study, we showed that barley flour exerts beneficial metabolic effects via the suppression of appetite and improvement of insulin sensitivity. These effects occur as a result of the promotion of gut hormone secretion following the production of gut microbial metabolite SCFAs from barley BG. These effects were abolished in GF mice and also observed in purified barley BG fed mice. These findings indicate that barley BG, as an indigestible polysaccharide, produce metabolic benefits by affecting host energy homeostasis via the production of SCFAs and likely other metabolites, induced by changes in the gut microbiota. This could represent a central mechanism underlying the effects of barley on metabolic improvements. Our results may contribute to the development of functional foods for the prevention of metabolic disorders, such as obesity and type 2 diabetes mellitus, through the selective breeding and development of barley containing higher levels of BG.

## Materials and methods

### Animals and diet

For HFD studies, 3-week-old male C57BL/6J mice were purchased from Japan SLC (Shizuoka, Japan). The male mice are well suited for the study of obesity-related metabolic dysfunction using an HFD feeding paradigm, because male mice gain weight to a greater extent compared to the females despite similar degrees of adipocyte hypertrophy [37]. C57BL/6J mice were housed in 12-h light-dark cycle under conventional conditions, and acclimated for 1 week with regular chow (CE-2, CREA, Tokyo, Japan). 4-week-old C57BL/6J mice were placed on a D12492 diet (60% kcal fat, Research diets, New Brunswick, NJ) or modified D12492 diet for 12 weeks (n = 8–13). The compositions of the diets are given in S1 and S2 Tables. The barley flour diet was adjusted so that the final percentages of protein, fat and carbohydrate were roughly equal. The barley flour group diet contains 0.6% (LBG; general barley) or 2% (HBG; Beau Fiber [38]) b-glucan in total diet. Purity and molecular weight of barley β-glucan in HFD is 92.5% and 125 kDa, respectively. In some experiments, 8-week-old male C57BL/6J mice were placed on a D12492 diet (60% kcal fat, Research diets) or modified D12492 diet for 2 weeks for the measurement of gut microbial composition. The composition of the diets was provided in S1 Table. All experimental procedures involving mice were performed according to protocols approved by the Committee on the Ethics of Animal Experiments of the Tokyo University of Agriculture and Technology (Permit Number: 28–87). For HFD studies under sterile conditions, male germ-free ICR mice were housed in vinyl isolators under a 12-h light-dark cycle. 4-week-old germ-free ICR mice were placed on a D12492 diet (50 kGy irradiated, Research diets) or modified D12492 diet (50 kGy irradiated, Research diets) for 12 weeks (n = 5). The composition of the diets was given in S1 Table. All experimental procedures involving germ free mice were performed according to protocols approved by the Institutional Animal Care and Use Committee of Keio University School of Medicine (permission No. 09036-(12)). For collection of blood and tissue samples, mice were sacrificed by anesthesia with somnopentyl. All efforts were made to minimize suffering.

### Biochemical analyses

At the end of the 12-week-experimental period, mice were sacrificed, and plasma samples were collected from inferior vena cava. The plasma glucose concentration was measured using One Touch Ultra Test Strips (LifeScan, Milpitas, CA). The plasma triglyceride concentration was measured by using LabAssay™ Triglyceride (Wako, Tokyo, Japan) following the manufacturer’s instructions. The concentrations of plasma PYY (Mouse/Rat PYY ELISA Kit, Wako), leptin (Mouse/Rat Leptin Quantikine ELISA Kit, R&D, Minneapolis, MN), GLP-1 (GLP-1 (Active) ELISA KIT, Shibayagi, Gunma, Japan), and insulin (Insulin ELISA KIT (RTU), Shibayagi) were determined by enzyme-linked immunosorbent assay (ELISA) as described previously [27]. For plasma GLP-1 measurement, plasma sample was treated with dipeptidyl peptidase IV (DPP-IV) inhibitor (Merck Millipore, Darmstadt, Germany) to prevent the degradation of active GLP-1.For glucose tolerance test (GTT), 24-h-fasted mice were given 1.5 mg of glucose per gram of body weight intraperitoneally (i.p.) or oral administration (p.o.) [18, 39, 40]. The plasma glucose concentration was monitored before injection and at 15, 30, 60, 90, and 120 min after injection. Basal glucose concentration at time 0 was set as 100% [41].For SCFAs measurement, SCFAs in feces were determined following a modified protocol as previously described [42]. Herein, the SCFA-containing ether layers were collected and pooled for GC-MS analysis using a GCMS-QP2010 Ultra (Shimadzu, Kyoto, Japan). The calibration curves for SCFAs were performed, and the concentration of SCFAs in each samples was evaluated over a specified concentration range.

### Quantification of fecal microbiota by real-time PCR

Fecal DNA was extracted from frozen samples using the FastDNA® SPIN Kit for Feces (MP Biomedicals, Santa Ana, CA) per the manufacturer’s instructions. Bacteria (*Bifidobacterium breve* JCM1192^T^, *Clostridium ramosum* JCM1298^T^, and *Bacteroides vulgatus* JCM5826^T^) were provided from Japan Collection of Microorganisms of RIKEN BRC and used as standards specifically for the DNA-based determination of fecal bacterial counts. Bacterial DNA was isolated using MonoFas Bacterial Genomic Kit IV (GLC science, Tokyo, Japan) following the manufacturer’s instructions. Quantitative real-time PCR analysis was performed with using SYBR Premix Ex Taq II (TaKaRa, Shiga, Japan) and the ABI7300 apparatus (Applied Biosystems, Foster City, CA). The reaction was performed at 95°C for 30 sec, followed by 40 cycles of 95°C for 5 sec, 58°C for 30 sec and 72°C for 1 min. The dissociation stage was analyzed at 95°C for 15 sec, followed by 1 cycle of 60°C for 1 min and 95°C for 15 sec. Standard curves for quantification consisted in ten-fold serial dilutions in the range of 10^8^ to 10^0^ copies of target 16S rRNA genes from each specific strains. Bacterial primer sequences are as follows; Firmicutes, 5’-GGAGYATGTGGTTTAATTCGAAGCA-3’ (forward) and 5’-AGCTGACGACAACCATGCAC-3’ (reverse); Bacteroidetes, 5’-CRAACAGGATTAGATACCCT-3’ (forward) and 5’-GGTAAGGTTCCTCGCGTAT-3’ (reverse); Actinobacteria, 5’-TACGGCCGCAAGGCTA-3’ (forward) and 5’-TCRTCCCCACCTTCCTCCG-3’ (reverse) [17, 43, 44].

### Statistical analysis

Values are presented as the mean ± S.E.M. Differences between groups were examined for statistical significance using Student’s t-test (two groups) or one-way analysis of variance followed by Tukey-Kramer’s post hoc test. P-values < 0.05 were considered statistically significant. We performed power analysis to justify the number of animals by using G*power analysis software.

## Supporting information

S1 Table Composition of Co, HBG and LBG diets. (DOC)

S2 Table Composition of HFC and HFB diets. (DOC)
