# Supplementary material for: Barley β-glucan improves metabolic condition via short-chain fatty acids produced by gut microbial fermentation in high fat diet fed mice
Source: PLoS One. 2018 Apr 26;13(4):e0196579. doi: 10.1371/journal.pone.0196579 (PMC5919537; doi:10.1371/journal.pone.0196579)
Supplement: S1 Table — (DOC) [file pone.0196579.s001.doc]

**S1 Table.** **Composition of Co, HBG and LBG diets.**

| Ingredient | Co | LBG | HBG |
| --- | --- | --- | --- |
|  |  | g |  |
| Casein | 200 | 183 | 184.5 |
| L-Cystine | 3 | 3 | 3 |
| Corn starch^2^ | 0 | 0 | 0 |
| Maltodextrin 10 | 125 | 92.9 | 100 |
| Sucrose | 68.8 | 0 | 0 |
| Cellulose, BW200 | 50 | 0 | 0 |
| Soybean oil | 25 | 25 | 25 |
| Lard | 245 | 241.7 | 239.8 |
| Mineral mix S10026^3^ | 10 | 10 | 10 |
| DiCalcium phosphate | 13 | 13 | 13 |
| Calcium carbonate | 5.5 | 5.5 | 5.5 |
| Potassium citrate, H_2_O | 16.5 | 16.5 | 16.5 |
| Vitamin mix V10001^4^ | 10 | 10 | 10 |
| Choline bitartrate | 2 | 2 | 2 |
| Low β-glucan Barley flour | 0 | 151 | 0 |
| High β-glucan Barley flour | 0 | 0 | 153 |
| Total weight, g | 773.8 | 753.6 | 762.3 |
|  |  |  |  |
| Energy, kcal/g | 5.1 | 5.3 | 5.2 |
| % Energy |  |  |  |
| Fat | 61 | 61 | 61 |
| Carbohydrate | 21 | 21 | 21 |
| Protein | 18 | 18 | 18 |
| % Weight |  |  |  |
| Carbohydrate | 26 | 27 | 27 |
| Fiber | 6.5 | 0 | 0 |
| Low β-glucan Barley flour | 0 | 20 | 0 |
| High β-glucan Barley flour | 0 | 0 | 20 |

^1^Prepared by Research Diet, Inc., New Brunswick, NJ.

^2^N-acetylcysteine was supplemented at 1 g/kg diet in replace of equal amount of corn starch.

^3^The mineral mix composition was as follows (amount in 10 g): 0.5 g Mg, 0.33 g S, 1.0 g Na, 1.6 g Cl, 1.6 mg Mo, 2.0 mg Cr, 6.0 mg Cu, 0.2 mg I, 37.0 mg Fe, 59 mg Mn, 0.16 mg Se and 29 mg Zn.

^4^The vitamin mix composition was as follows (amount in 10 g): 4000 IU vitamin A palmitate, 1,000 IU vitamin D3, 50 IU vitamin E acetate, 0.5 mg menadione sodium bisulfite, 0.2 mg biotin, 10 μg cyanocobalamin, 2 mg folic acid, 30 mg nicotinic acid, 16 mg calcium pantothenate, 7 mg pyridoxine-HCl, 6 mg riboflavin and 6 mg thiamin HCl.

^5^The barley flour group diet contains 0.6% (LBG; general barley) or 2-4% (HBG; Beau Fiber) b-glucan in total diet.

Co, Control; HBG, β-glucan rich barley; LBG, general barley.
